# Laparoscopic gastrectomy plus D2 lymphadenectomy is as effective as open surgery in terms of long-term survival: a single-institution study on gastric cancer

**DOI:** 10.1186/s12957-021-02218-1

**Published:** 2021-04-07

**Authors:** Yawei Wang, Yan Wang, Wang Wu, Xiaofang Lu, Tailai An, Jiling Jiang

**Affiliations:** 1 The First Department of Surgery, Shenzhen Traditional Chinese Medicine Hospital, Fuhua Road 1, Futian District, Shenzhen, 518020 Guangdong China; 2 Department of Radiology, Shenzhen People’s Hospital, Dongmen North Road 1017, Luohu District, Shenzhen, 518020 Guangdong China; 3 Center of Digestive Diseases, The Seventh Affiliated Hospital, Sun Yat-sen University, Zhenyuan Road 628, Guangming District, Shenzhen, 518107 Guangdong China; 4 Department of Pathology, The Seventh Affiliated Hospital, Sun Yat-sen University, Zhenyuan Road 628, Guangming District, Shenzhen, 518107 Guangdong China

**Keywords:** Gastric cancer, Laparoscopic gastrectomy plus D2 lymphadenectomy, Overall survival, Disease-free survival, Positive lymph node ratio

## Abstract

**Background:**

Laparoscopic surgery has been widely accepted to treat early-stage gastric cancer. However, it is still controversial to perform laparoscopic gastrectomy plus D2 lymphadenectomy for locally advanced gastric cancer. We performed the present study to compare the long-term outcomes of patients after laparoscopic or open gastrectomy plus D2 lymphadenectomy.

**Methods:**

The clinicopathological data of 182 gastric cancer patients receiving gastrectomy plus D2 lymphadenectomy between January 2011 and December 2015 at Shenzhen Traditional Chinese Medicine Hospital were retrospectively retrieved. The overall survival (OS) and disease-free survival (DFS) of these 182 patients were compared. Then, the prognostic significance of positive lymph node ratio (LNR) was assessed.

**Results:**

As a whole, OS (*P* = 0.789) and DFS (*P* = 0.672) of patients receiving laparoscopic gastrectomy plus D2 lymphadenectomy were not significantly different from those of patients receiving open surgery. For stage I patients, laparoscopic gastrectomy plus D2 lymphadenectomy was not significantly different from open surgery in terms of OS (*P* = 0.573) and DFS (*P* = 0.157). Similarly, for stage II patients, laparoscopic gastrectomy plus D2 lymphadenectomy was not significantly different from open surgery in terms of OS (*P* = 0.567) and DFS (*P* = 0.830). For stage III patients, laparoscopic gastrectomy plus D2 lymphadenectomy was not significantly different from open surgery in terms of OS (*P* = 0.773) and DFS (*P* = 0.404). Laparoscopic or open gastrectomy plus D2 lymphadenectomy was not proven by Cox regression analysis to be an independent prognostic factor for OS and DFS. High LNR was significantly associated with worse OS (*P* < 0.001) and DFS (*P* < 0.001). Surgical type did not significantly affect prognosis of patients with low LNR or survival of patients with high LNR.

**Conclusions:**

For patients with gastric cancer, laparoscopic gastrectomy plus D2 lymphadenectomy was not inferior to open surgery in terms of long-term outcomes. LNR is a useful prognostic marker for GC patients.

## Background

Globally, gastric cancer (GC) is one of the most common cancers with one of the highest mortality rates, especially in China as almost a half of gastric cancer patients are diagnosed in China [1, 2]. Survival of GC patients has been remarkably improved over the past few decades due to the wide application of multidisciplinary teamwork (MDT). Curative gastrectomy plus D2 lymphadenectomy remains the cornerstone of this MDT mode despite the introduction of targeted therapy and immunotherapy.

For patients with early-stage GC, laparoscopic gastrectomy has become the preferred choice given its similar long-term oncological outcomes and significantly better short-term outcomes [3–6]. It was reported by the Korean Laparoendoscopic Gastrointestinal Surgery Study (KLASS) group that laparoscopic gastrectomy was related with much better short-term outcomes such as less blood loss, less severe postoperative pain, faster recovery, and much shorter hospital stay and similar long-term oncological outcomes [7]. In a multicenter randomized clinical trial published in 2020, similar conclusions were drawn [5]. Thus, as far as we are concerned, in most guidelines and multicenter clinical trial, laparoscopic gastrectomy is recommended as the treatment of choice for early-stage GC.

Unlike early-stage gastric cancer, surgical choice for locally advanced GC still remains controversial although a few multicenter clinical trials have been carried out. Concerns from surgeons include trocar-site tumor seeding [8], technical difficulties in en-bloc removal of cancerous tissues, and inadequate lymphadenectomy. However, some multicenter clinical trials supporting the application of laparoscopic gastrectomy for locally advanced gastric cancer have been published. Jiang Yu et al. reported that for locally advanced GC, laparoscopic gastrectomy was not inferior to traditional open gastrectomy in terms of 3-year disease-free survival [9, 10]. Similarly, the Korean Laparoendoscopic Gastrointestinal Surgery Study (KLASS) group reported that for patients with locally advanced GC, laparoscopic distal gastrectomy with D2 lymphadenectomy was similar to open surgery regarding relapse-free survival, suggesting that laparoscopic distal gastrectomy with D2 lymphadenectomy has the potential as the standard treatment for locally advanced GC [11]. As for short-term outcomes, Hyuk-Joon Lee et al. reported that for locally advanced GC, laparoscopic distal gastrectomy with D2 lymphadenectomy was related with lower morbidity rate, quicker recovery, and less severe postoperative pain [12]. Despite these multicenter clinical trials, it is still necessary for us to perform studies to further clarify the roles of laparoscopic gastrectomy in locally advanced GC since some shortcomings of these clinical trials are not to be neglected. Firstly, in these studies, only relapse-free survival or disease-free survival is compared while overall survival has not been covered. Secondly, follow-ups in these studies are rather short, which limits our assessment of long-term survival. Thirdly, only distal GC are studied, making it difficult for us to evaluate the appropriateness of laparoscopic gastrectomy for proximal GC.

Given the aforementioned evidences, we hypothesized that laparoscopic gastrectomy plus D2 lymphadenectomy was not inferior to open surgery in terms of long-term outcomes (both OS and DFS). Thus, we performed the present study with the aim of comparing laparoscopic gastrectomy plus D2 lymphadenectomy with open surgery in terms of long-term outcomes.

## Methods

### Study population

**Fig. 1:**
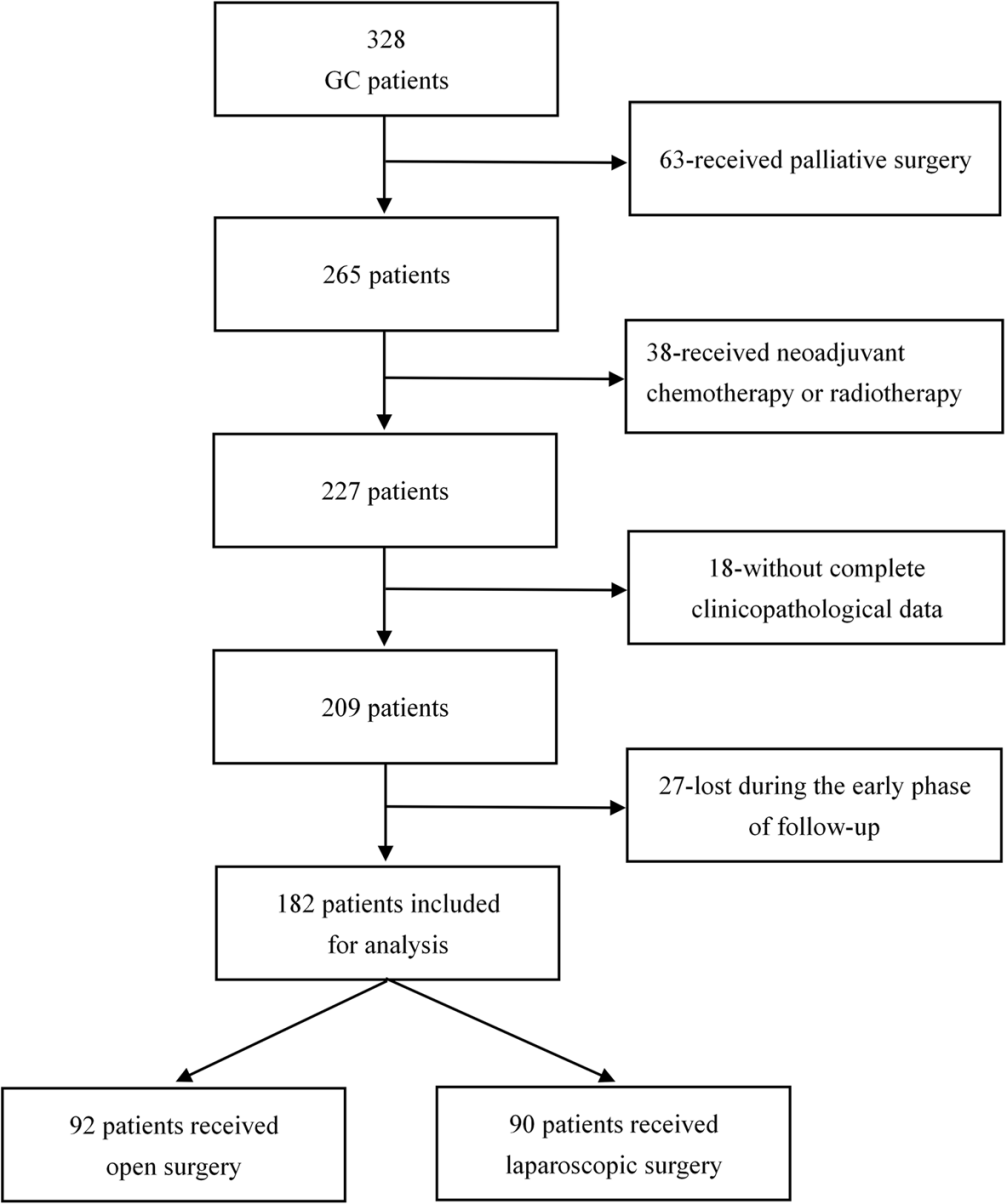
Flowchart illustrating the detailed screening process

Clinicopathological data of GC patients receiving open or laparoscopic surgery between January 2012 and December 2015 were retrospectively collected through screening the medical records. Initially, a total of 328 GC patients had undergone surgery at Shenzhen Traditional Chinese Medicine Hospital. The following criteria were adopted to exclude unqualified patients: receiving palliative surgery, receiving neoadjuvant chemotherapy or radiotherapy, without complete clinicopathological data, and lost during the early phase of follow-up. The detailed screening process was demonstrated in Fig. 1. This study obtained ethical approval from the Ethics Committee of Shenzhen Traditional Medicine Hospital. All the patients had given their written informed consents. The whole process of the present study was in accordance with Declaration of Helsinki [13].

### Cancer staging and grading

Pathological stages of these patients were re-determined according to the eighth edition of the American Joint Committee on Cancer (AJCC)/International Union Against Cancer TNM classification system. The Borrmann classification is as follows: Type I: polypoid type, Type II: locally ulcerative type, Type III: infiltrating ulcerative type, and Type IV: diffusely infiltrating type. In terms of differentiation, gastric cancer can be graded using the following criteria: Gx: grade cannot be assessed, G1: well differentiated, G2: moderately differentiated, and G2: poorly differentiated or undifferentiated.

### Choosing the most suitable patients for laparoscopic surgery

Firstly, history of upper abdominal surgery contradicts laparoscopic surgery as adhesion might make the surgeons unable to safely perform the designated laparoscopic surgery. Secondly, laparoscopic surgery would not be performed if patients refused the chief surgeon’s recommendation. Thirdly, if the chief surgeon thought the extensive lymph node metastasis or primary GC lesions could not be radically and safely dissected via laparoscopy, then laparoscopic gastrectomy would not be performed. Additionally, for each individual patient, pulmonary function test, liver function test, kidney function test, and cardiac function test were routinely performed to exclude those suffering from serious dysfunction of the aforementioned organs.

### Surgical procedures

All the laparoscopic or open gastrectomy plus D2 lymphadenectomy for GC in our department complied with principles of the excision extensions suggested by Chinese and Japanese guidelines [4]. All the surgical procedures of the 182 GC patients were accomplished by the director of our department, Professor Jiling Jiang, suggesting that the surgery-related bias of this study was minimal. During laparoscopic surgery, five trocars were inserted for diagnostic cancer staging and lymph node dissection and a minilaparotomy for specimen retraction and anastomosis. Ultrasonic scalpel was used for mobilization and dissection in both open and laparoscopic surgeries. En-bloc removal of primary tumor and metastatic lymph nodes was performed for all the patients. For distal GC, distal gastrectomy plus D2 lymphadenectomy was performed. For proximal GC, total gastrectomy plus D2 lymphadenectomy was applied. Proximal gastrectomy was seldomly performed at our hospital as the incidence of esophageal reflux was rather high. The way to perform gastroenterostomy was determined by surgeons’ choice and local anatomy.

### Postoperative adjuvant therapy

According to the Chinese guideline on GC, patients with stage I GC are not recommended to undergo postoperative chemotherapy, while those with stage II (T1-2N1-3M0, T3-4N0M0) and stage III (T3-4aN1-3M0) are suggested to receive postoperative chemotherapy. Due to the lack of radiotherapy equipment, postoperative radiotherapy is not widely applied in our hospital. Additionally, some patients did not receive postoperative chemotherapy as instructed. Thus, in the present study, a total of 81 patients received postoperative chemotherapy. Eight patients (3 in the laparoscopic group (LG) and 5 in the open group (OG)) took S1 orally. XELOX regimen was applied to 35 patients (20 in the LG and 15 in the OG). SOX regimen was applied to 38 patients (16 in the LG and 22 in the OG).

### Follow-up plan and clinical outcomes

All the patients were advised to participate in a follow-up after the surgery unless otherwise contraindicated. The follow-up plan included the following: physical examination every 3 months for the first 2 years and every 6 months thereafter, detection of carcinoembryonic antigen and cancer antigen 19-9 every 3 months for the first 2 years and every 6 months thereafter, abdominal computed tomographic scans every 6 months for 3 years, and annual upper gastrointestinal endoscopy for 3 years. Recurrence was diagnosed by combining medical history, physical examination, radiological examination, and pathological examination (cytological or histological, histological is preferred when possible). Positron emission tomography–computed tomography (PET-CT) was performed if local or distant recurrence was suspected. Overall survival (OS) was defined as the duration between surgery and the date of death. Disease-free survival (DFS) is defined as the time length between surgery and the date of cancer recurrence or death (whichever occurred first).

### Prognostic significance of positive lymph node ratio

As multiple studies have reported that positive lymph node ratio is significantly associated with prognosis of GC patients, we thus performed further analysis to verify its prognostic significance among GC patients in our study. Metastatic lymph nodes to dissected lymph nodes ratio was defined as positive lymph node ratio (LNR). Initially, receiver operating characteristics (ROC) analysis was accomplished to identify the optimal cutoff value for LNR. Then, Kaplan-Meier analyses were performed to assess the effects of LNR on OS and DFS of the 182 GC patients included in our study. Additionally, we further performed Kaplan-Meier analysis to testify whether patients with high or low LNR would have significantly different prognosis after undergoing laparoscopic or open surgery.

### Statistical analysis

By operation type (laparoscopic or open), patients were divided into the open group (OG) or laparoscopic group (LG). Clinicopathological variables of the two groups were compared by chi-square test and Fisher’s exact test. The Kaplan-Meier method was utilized to calculate and compare survival of patients in the two groups, which was then tested via log-rank test. Univariate Cox regression analysis was performed to determine variables that were significantly associated with OS or RFS. Then, these variables proven by univariate Cox regression analysis to be significantly associated with OS or RFS were included in multivariate Cox regression analysis to identify independent prognostic factors for OS or RFS. Then, we accomplished subgroup analysis to evaluate whether operation types would influence survival of patients with cancer of different stages. SPSS 22 (Chicago, IL, USA) was used to perform all the statistical analyses. The tests performed in the present study were two-sided and a *P* value < 0.05 was defined as statistically significant.

## Results

### Baseline clinicopathological characteristics

From January 2011 and December 2015, a total of 328 GC patients underwent an operation at Shenzhen Traditional Chinese Medicine Hospital. After being screened under exclusive criteria, 146 ones of these 328 patients were excluded, leaving 182 qualified patients for this study. The flowchart illustrating the screening procedure was demonstrated in Fig. 1. Of the 182 patients, 92 ones received open gastrectomy plus D2 lymphadenectomy while 90 ones underwent laparoscopic surgery. Baseline clinicopathological data of these 182 GC patients were summarized and demonstrated in Table 1.

**Table 1:** Clinicopathological characteristics of patients receiving open or laparoscopic surgery

Characteristics	No.	Operation type		*χ*^2^/*t* value	*P* value
		Open (*N* = 92)	Laparoscopic (*N* = 90)		
Age		57.45 ± 10.43	58.07 ± 13.37	− 0.350	0.727
≤ 60years	107 (58.8%)	57 (62.0%)	50 (55.6%)	0.769	0.380
> 60years	75 (41.2%)	35 (38.0%)	40 (44.4%)		
Gender					
Male	134 (73.6%)	70 (76.1%)	64 (71.1%)	0.580	0.446
Female	48 (26.4%)	22 (23.9%)	26 (28.9%)		
Tumor size					
≤ 5 cm	132 (72.5%)	70 (76.1%)	62 (68.9%)	1.183	0.277
> 5 cm	50 (27.5%)	22 (23.9%)	28 (31.1%)		
Borrmann classification					
I+II	69 (37.9%)	34 (37.0%)	35 (38.9%)	0.072	0.788
III+IV	113 (62.1%)	58 (63.0%)	55 (61.1%)		
Histological differentiation					
Well	5 (2.8%)	3 (3.3%)	2 (2.2%)	1.459	0.482
Moderate	49 (26.9%)	28 (30.4%)	21 (23.3%)		
Poor	128 (70.3%)	61 (66.3%)	67 (74.5%)		
Vascular invasion					
No	152 (83.5%)	76 (82.6%)	76 (84.4%)	0.111	0.739
Yes	30 (16.5%)	16 (17.4%)	14 (15.6%)		
Nerve invasion					
No	167 (91.8%)	81 (88.0%)	86 (95.6%)	3.395	0.065
Yes	15 (8.2%)	11 (12.0%)	4 (4.4%)		
Depth of invasion					
T1	29 (15.9%)	14 (15.2%)	15 (16.7%)	4.154	0.386
T2	24 (13.2%)	11 (12.0%)	13 (14.4%)		
T3	70 (38.5%)	31 (33.7%)	39 (43.3%)		
T4a	38 (20.9%)	24 (26.1%)	14 (15.6%)		
T4b	21 (11.5%)	12 (13.0%)	9 (10.0%)		
Lymph node metastasis					
N0	73 (40.1%)	35 (38.0%)	38 (42.2%)	0.859	0.930
N1	34 (18.7%)	17 (18.5%)	17 (18.9%)		
N2a	33 (18.1%)	19 (20.7%)	14 (15.6%)		
N2b	24 (13.2%)	12 (13.0%)	12 (13.3%)		
N3	18 (9.9%)	9 (9.8%)	9 (10.0%)		
pTNM					
I	43 (23.6%)	21 (22.8%)	22 (24.4%)	2.081	0.353
II	57 (31.3%)	25 (27.2%)	32 (35.6%)		
III	82 (45.1%)	46 (50.0%)	36 (40.0%)		
CEA level(μg/L)					
≤ 5	160 (87.9%)	74 (80.4%)	86 (95.6%)	9.788	0.002
> 5	22 (12.1%)	18 (19.6%)	4 (4.4%)		
Resection range					
Proximal	2 (1.1%)	2 (2.2%)	0 (0%)	3.403	0.182
Distal	94 (51.6%)	43 (46.7%)	51 (56.7%)		
Total	86 (47.3%)	47 (51.1%)	39 (43.3%)		
Lymphadenectomy					
D2	158 (86.8%)	79 (85.9%)	79 (87.8%)	0.145	0.704
D2+	24 (13.2%)	13 (14.1%)	11 (12.2%)		
NORLN		33.73 ± 17.32	31.29 ± 13.89	1.047	0.297
NOMLN		5.03 ± 8.50	4.83 ± 8.56	0.158	0.875
PHS (days)		11.97 ± 6.76	9.14 ± 2.45	3.728	0.000
Operation time (min)		249.87 ± 48.79	307.85 ± 64.99	6.795	0.000

*CEA* carcinoembryonic antigen, *NORLN* number of retrieved lymph nodes, *NOMLN* number of metastatic lymph nodes, *PHS* postoperative hospital stay

### Survival analysis

**Fig. 2:**
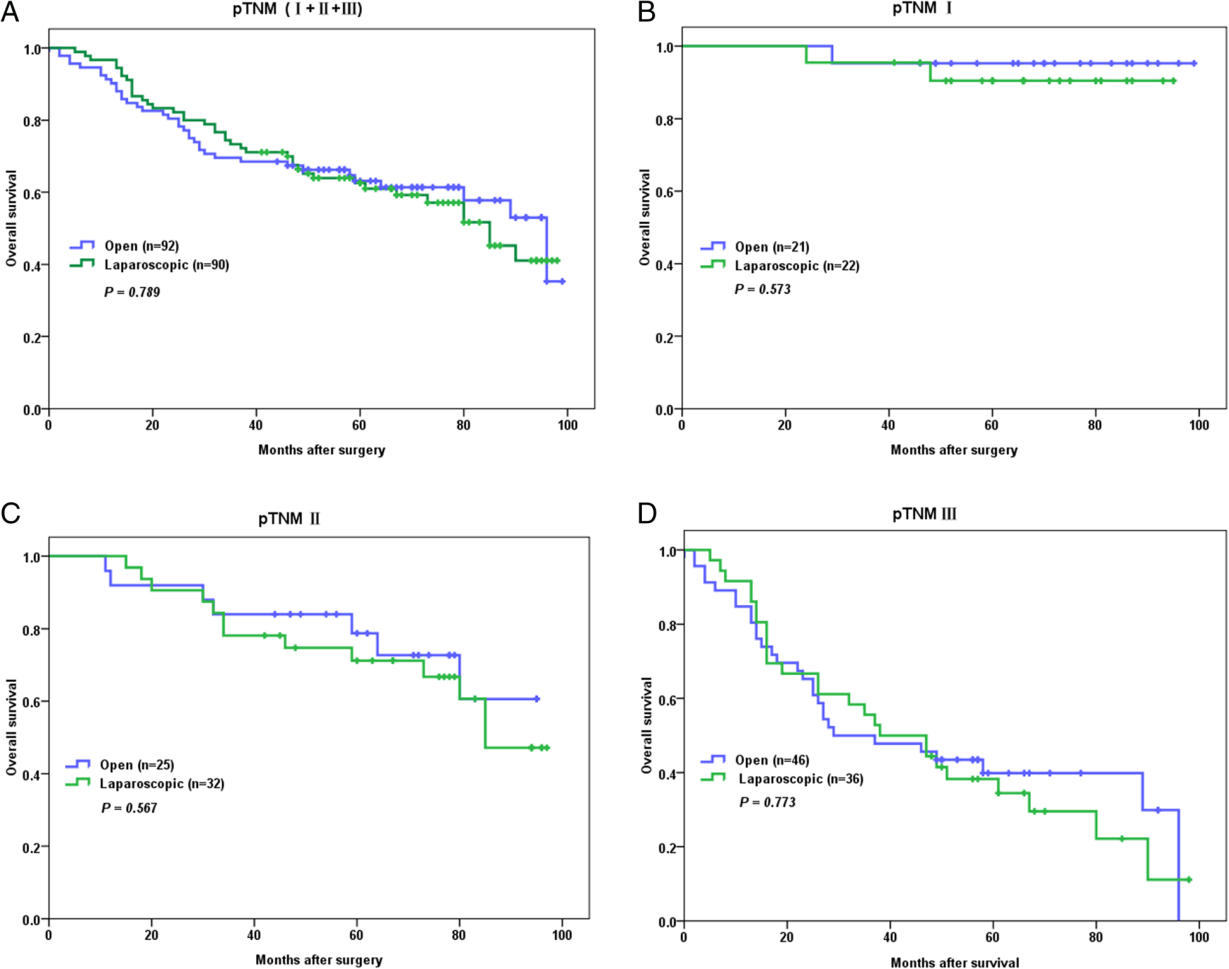
Kaplan-Meier estimates of overall survival according to operation type. **a** Overall survival of all the GC patients (LG vs OG). **b** Overall survival of stage I GC patients. **c** Overall survival of stage II GC patients. **d** Overall survival of stage III GC patients

**Fig. 3:**
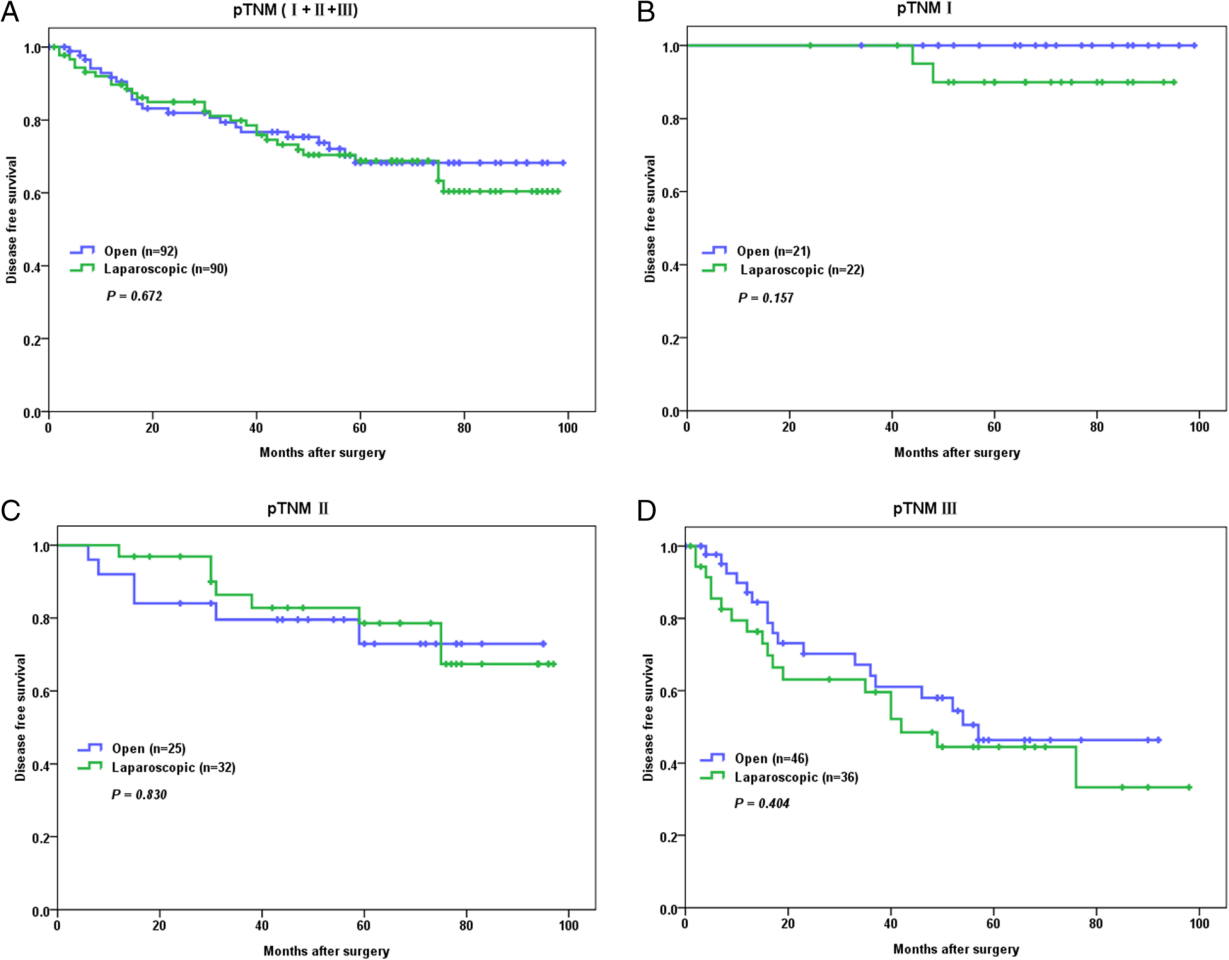
Kaplan-Meier estimates of disease-free survival according to operation type. **a** Disease-free survival of all the GC patients. **b** Disease-free survival of stage I GC patients. **c** Disease-free survival of stage II GC patients. **d** Disease-free survival of stage III GC patients

The median follow-up of the 182 patients was 42 months (0 to 99 months). Comparisons between OG and LG in terms of OS and DFS of the 182 patients were made, revealing that OG and LG were not significantly different from each other regarding either OS (*P* = 0.789) (Fig. 2a) or DFS (*P* = 0.672) (Fig. 3a).

Then, subgroup analyses were performed. For patients with stage I GC, laparoscopic gastrectomy plus D2 lymphadenectomy was not significantly different from open surgery in terms of OS (*P* = 0.573) (Fig. 2b) and DFS (*P* = 0.157) (Fig. 3b). For patients with stage II GC, it was also revealed that laparoscopic gastrectomy plus D2 lymphadenectomy was not any different from open surgery in terms of OS (*P* = 0.567) (Fig. 2c) and DFS (*P* = 0.830) (Fig. 3c). Similarly for patients with stage III GC, it was demonstrated that laparoscopic gastrectomy plus D2 lymphadenectomy was not significantly different from open surgery in terms of OS (*P* = 0.773) (Fig. 2d) and DFS (*P* = 0.404) (Fig. 3d).

Evaluation of the long-term outcomes of the whole population was also performed. It was revealed through univariate Cox regression analysis that gender (*P* = 0.029, HR = 1.711, 95% CI: 1.057–2.772), tumor size (*P* < 0.001, HR = 2.318, 95% CI: 1.471–3.655), Bormmann classification (*P* < 0.001, HR = 3.786, 95% CI: 2.121–6.758), histological differentiation (*P* = 0.019, HR = 1.817, 95% CI: 1.104–2.990), depth of invasion (*P* < 0.001, HR = 1.875, 95% CI: 1.524–2.306), lymph node metastasis (*P* < 0.001, HR = 2.183, 95% CI: 1.821–2.617), pTNM (*P* < 0.001, HR = 3.459, 95%CI: 2.338–5.119), vascular invasion (*P* < 0.001, HR = 3.420, 95% CI: 2.066–5.661), nerve invasion (*P* = 0.005, HR = 2.611, 95% CI: 1.328–5.135), carcinoembryonic antigen (CEA; *P* = 0.006, HR = 2.255, 95% CI: 1.261–4.032), number of metastatic lymph nodes (*P* < 0.001, HR = 1.090, 95%CI: 1.070–1.110), and resection range (*P* = 0.029, HR = 1.625, 95% CI: 1.052–2.510) were significantly associated with OS (Table 2). Then, these variables proven by univariate Cox regression analysis to be significantly associated with OS were included in multivariate Cox regression analysis, results of which demonstrated that Bormmann classification (*P* = 0.014, HR = 2.252, 95% CI: 1.175–4.316), lymph node metastasis (*P* = 0.036, HR = 1.483, 95% CI: 1.296–1.511), pTNM (*P* = 0.027, HR = 2.379, 95% CI: 1.968–2.549), and number of metastatic lymph nodes (*P* = 0.032, HR = 1.052, 95% CI: 1.004–1.101) were independent prognostic factors for OS (Table 2).

**Table 2:** Cox proportional-hazard regression analysis for overall survival

Characteristics	Univariate analysis				Multivariate analysis			
	*P* value	HR	95.0% CI for Exp(B)		*P* value	HR	95.0% CI for Exp(B)	
			Lower	Upper			Lower	Upper
Gender	0.029	1.711	1.057	2.772				
Age	0.409	1.207	0.772	1.888				
Tumor size	0.000	2.318	1.471	3.655				
Borrmann classification	0.000	3.786	2.121	6.758	0.014	2.252	1.175	4.316
Histological differentiation	0.019	1.817	1.104	2.990				
Depth of invasion	0.000	1.875	1.524	2.306				
Lymph node metastasis	0.000	2.183	1.821	2.617	0.036	1.483	1.296	1.511
pTNM	0.000	3.459	2.338	5.119	0.027	2.379	1.968	2.549
Vascular invasion	0.000	3.420	2.066	5.661				
Nerve invasion	0.005	2.611	1.328	5.135				
CEA	0.006	2.255	1.261	4.032				
NORLN	0.522	1.004	0.991	1.018				
NOMLN	0.000	1.090	1.070	1.110	0.032	1.052	1.004	1.101
Resection range	0.029	1.625	1.052	2.510				
Lymphadenectomy	0.180	1.527	0.823	2.832				
PHS	0.647	1.009	0.970	1.050				
Operation time	0.081	1.003	1.000	1.007				

*CEA* carcinoembryonic antigen, *NORLN* number of retrieved lymph nodes, *NOMLN* number of metastatic lymph nodes, *PHS* postoperative hospital stay

Similarly, by univariate Cox regression analysis, it was revealed that tumor size (*P* = 0.004, HR = 2.295, 95%CI: 1.311–4.018), Bormmann classification (*P* = 0.002, HR = 2.814, 95%CI: 1.475–5.370), histological differentiation (*P* = 0.008, HR = 2.515, 95% CI: 1.266–4.999), depth of invasion (*P* < 0.001, HR = 1.848, 95% CI: 1.443–2.365), lymph node metastasis (*P* < 0.001, HR = 2.045, 95%CI: 1.645–2.542), pTNM (*P* < 0.001, HR = 3.431, 95% CI: 2.419–5.479), vascular invasion (*P* < 0.001, HR = 4.729, 95% CI: 2.618–8.542), nerve invasion (*P* = 0.006, HR = 2.924, 95% CI: 1.364–6.269), CEA (*P* = 0.048, HR = 2.066, 95% CI: 1.006–4.243), and number of metastatic lymph nodes (*P* < 0.001, HR = 1.097, 95% CI: 1.069–1.126) were significantly associated with DFS (Table 3). Then, these variables proven by univariate Cox regression analysis to be significantly associated with DFS were enrolled in multivariate Cox regression analysis, results of which demonstrated that histological differentiation (*P* = 0.039, HR = 2.198, 95% CI: 1.040–4.644), pTNM (*P* = 0.019, HR = 3.778, 95% CI: 2.561–4.182), vascular invasion (*P* = 0.007, HR = 2.687, 95% CI: 1.311–5.506), and number of metastatic lymph nodes (*P* = 0.047, HR = 1.060, 95% CI: 1.001–1.123) were independent prognostic factors for DFS (Table 3).

**Table 3:** Cox proportional-hazard regression analysis for disease-free survival

Characteristics	Univariate analysis				Multivariate analysis			
	*P* value	HR	95.0% CI for Exp(B)		*P* value	HR	95.0% CI for Exp(B)	
			Lower	Upper			Lower	Upper
Gender	0.103	1.623	0.907	2.906				
Age	0.710	0.899	0.514	1.573				
Tumor size	0.004	2.295	1.311	4.018				
Borrmann classification	0.002	2.814	1.475	5.370				
Histological Differentiation	0.008	2.515	1.266	4.999	0.039	2.198	1.040	4.644
Depth of invasion	0.000	1.848	1.443	2.365				
Lymph node metastasis	0.000	2.045	1.645	2.542				
pTNM	0.000	3.431	2.149	5.479	0.019	3.778	2.561	4.182
Vascular invasion	0.000	4.729	2.618	8.542	0.007	2.687	1.311	5.506
Nerve invasion	0.006	2.924	1.364	6.269				
CEA	0.048	2.066	1.006	4.243				
NORLN	0.288	1.009	0.992	1.026				
NOMLN	0.000	1.097	1.069	1.126	0.047	1.060	1.001	1.123
Resection range	0.208	1.400	0.829	2.362				
Lymphadenectomy	0.055	1.971	0.986	3.941				
PHS	0.334	1.022	0.978	1.069				
Operation time	0.472	1.002	0.997	1.006				

*CEA* carcinoembryonic antigen, *NORLN* number of retrieved lymph nodes, *NOMLN* number of metastatic lymph nodes, *PHS* postoperative hospital stay

### Prognostic significance of LNR among GC patients

**Fig. 4:**
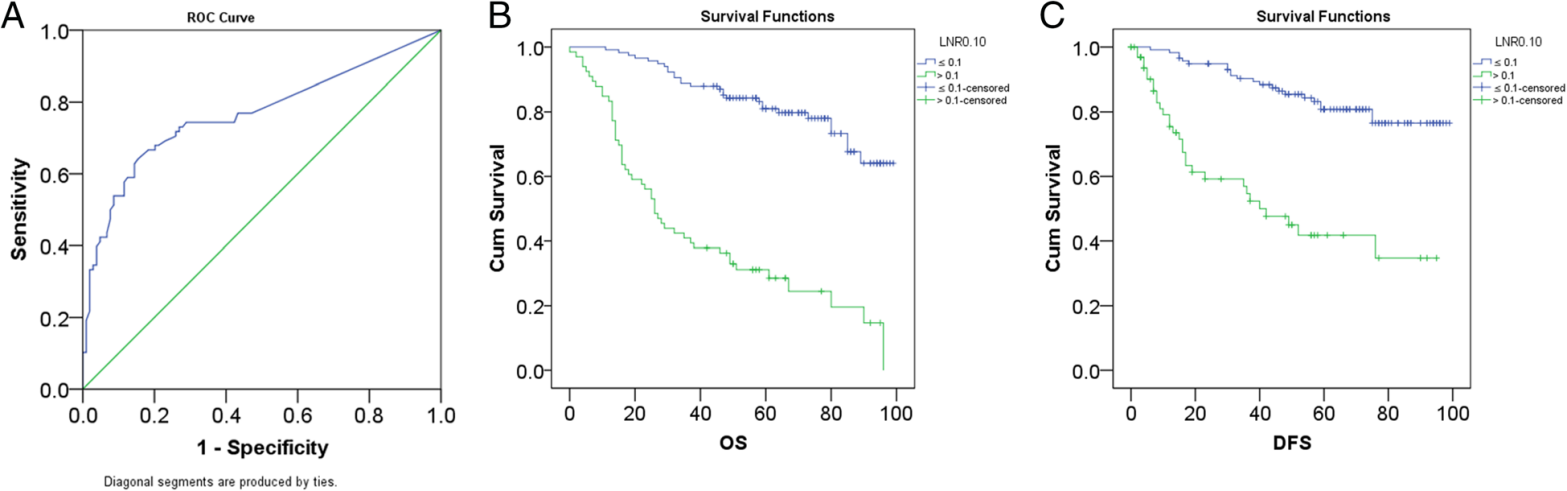
Identification of the optimal cutoff value for LNR and comparison between patients with low LNR and those with high LNR in terms of OS and DFS. **a** The identification of the optimal cutoff value of LNR by ROC analysis. **b** High LNR was significantly associated with worse OS. **c** High LNR was significantly associated with worse DFS

**Fig. 5:**
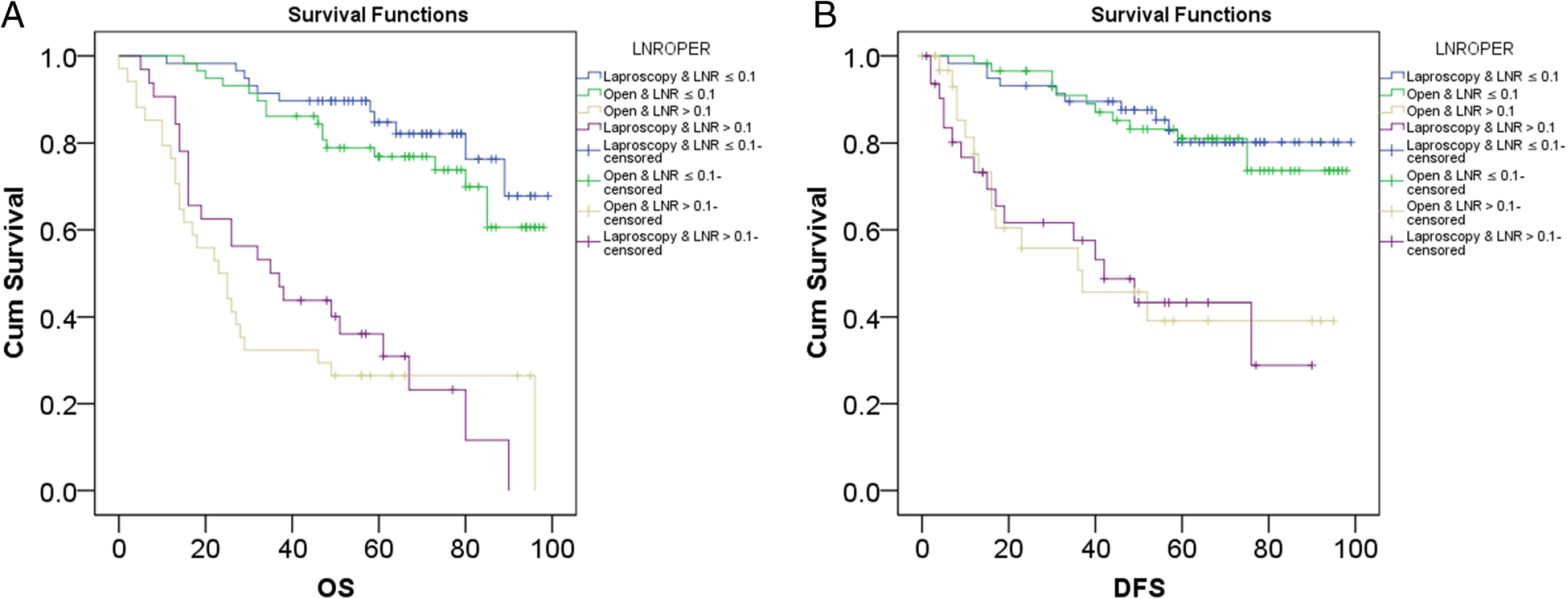
The effects of surgical type on prognosis of patients with low or high LNR. **a** Surgical type did not affect OS of patients with low or high LNR. **b** Surgical type did not affect DFS of patients with low or high LNR

Initially, it was revealed by ROC analysis that the optimal cutoff value for LNR was 0.1 at which area under curve of ROC curve was the biggest (AUC = 0.767) (Fig. 4a). Then, patients were divided into the high-LNR group and low-LNR group. As revealed by Kaplan-Meier analysis, LNR was significantly associated with worse OS (*P* < 0.001) (Fig. 4b) and DFS (*P* < 0.001) (Fig. 4c). Similarly, through Kaplan-Meier analysis, it was demonstrated that laparoscopic gastrectomy was not significantly different from open surgery regarding OS of patients with low LNR (Fig. 5a). In a similar way, we also demonstrated that laparoscopic gastrectomy was not significantly different from open surgery regarding OS of patients with high LNR (Fig. 5a). As for DFS of patients with low LNR, laparoscopic gastrectomy was not significantly from open surgery (Fig. 5b). Likewise, laparoscopic gastrectomy was not significantly different from open surgery regarding DFS of patients with high LNR (Fig. 5b).

## Discussion

The retrospective study conducted at our hospital demonstrated that laparoscopic gastrectomy plus D2 lymphadenectomy was not significantly different from open surgery regarding long-term outcomes of GC patients. This discovery was not only proven among all the patients (stages I, II, and III) but also among patients at each stage. Operation type was not proven by Cox regression analysis to be an independent predictive factor for either OS or RFS.

From the technical perspective, laparoscopic surgery was superior to open surgery in terms of visualization, exposure, and manipulations of organs, blood vessels, and nerves. Despite the established safety of laparoscopic surgery regarding short-term outcomes, the efficiency of laparoscopic gastrectomy plus D2 lymphadenectomy in terms of long-term outcomes is still worth being further investigated. Unlike open surgery, during laparoscopic surgery, en-bloc removal of primary tumor and adequate D2 lymphadenectomy may be compromised. Cancerous tissue manipulation and pneumoperitoneum established during laparoscopic surgery could cause dissemination of cancer cells, particularly for serosa-invading and lymph node-positive gastrointestinal malignant tumors [14–17], thus potentially increasing the possibility of cancer recurrence [14], which, however, has not been observed in previous studies and our study. A few clinical trials have confirmed the safety of laparoscopic gastrectomy plus D2 lymphadenectomy in treating GC [12, 18, 19]. The short-term advantages and oncological safety of laparoscopic surgery for stage I GC has recently been confirmed by one Korean randomized trial and Chinese clinical study, demonstrating decreased morbidity rates and at least non-inferior long-term outcomes related with laparoscopic approach [5, 7]. Oncological safety of laparoscopic gastrectomy plus D2 lymphadenectomy for locally advanced gastric cancer (most were stages II and III) has previously been proven by a few large randomized clinical trials [9, 12, 20]. In these studies and other reports, it was reported that in comparison with open surgery, laparoscopic gastrectomy plus D2 lymphadenectomy was related with significantly lower intraoperative and postoperative complication rates and quicker recovery [5, 7, 9, 12, 20]. However, for stage I GC, a D2 lymphadenectomy is not always indicated and concerns about risks and benefits of laparoscopic gastrectomy plus D2 lymphadenectomy have not been fully resolved [5, 7, 21]. Additionally, for this study, despite the fact, we have confirmed similar long-term outcomes between laparoscopic surgery and open surgery, comparisons of them regarding short-term outcomes have not been fully made, which was one of the limits of this study. Therefore, we could conclude that laparoscopic gastrectomy plus D2 lymphadenectomy could be safely performed by experienced surgeons given its similar long-term outcomes to open surgery and significantly better short-term outcomes, which is still needed to be validated by more randomized clinical trials as in these aforementioned clinical trials the follow-up period was quite short.

As was mentioned above, in the present study, it was revealed that OS of GC patients receiving laparoscopic gastrectomy plus D2 lymphadenectomy was not inferior to that of GC patients undergoing open surgery. Furthermore, according to previous multicenter randomized clinical trials comparing short-term outcomes of GC patients receiving these two surgical approaches, laparoscopic gastrectomy plus D2 lymphadenectomy was significantly superior to open surgery [5, 7]. In the present study, although we have not fully compared short-term outcomes of GC patients receiving these two types of surgery, we have revealed that laparoscopic surgery is related with much shorter hospital stay, which is consistent with previous studies [5, 7]. In this study, other variables indicating short-term outcomes such as blood loss, postoperative pain, and recovery situation have not been explored, which was one of the limitations of this study. We have also compared the number of retrieved lymph nodes between these two surgical approaches, results of which demonstrated that these two surgical approaches were not significantly different from each other in terms of the number of retrieved lymph nodes. Studies comparing laparoscopic surgery with open surgery in terms of retrieved lymph nodes have been previously published. Yu J et al. reported that the number of retrieved lymph nodes in laparoscopic gastrectomy plus D2 lymphadenectomy was not significantly different from that in open surgery [9]. Lee HJ et al. also reported that no significant difference was observed between laparoscopic gastrectomy plus D2 lymphadenectomy and open surgery in terms of retrieved lymph nodes [12]. Thus, we could conclude that adequate lymph nodes could be retrieved as long as the surgery is performed by a capable surgeon. In this study, we also found that operation time of laparoscopic surgery was significantly longer than that of open surgery, which was consistent with previous studies. Kodera Y et al. reported that it took significantly longer to perform laparoscopic total gastrectomy for gastric cancer than to perform an open surgery [22]. Similarly, it was reported by Katai H that it took much longer time to perform laparoscopy-assisted gastrectomy with nodal dissection for stage IA and IB gastric cancers [23]. The conclusion that the operation time of laparoscopic gastrectomy was much longer than that of open gastrectomy was also drawn in a study by Best et al. [24]. However, we speculate this difference in operation time of two surgical approaches is caused by a learning curve effect [25] and we believe that with the accumulation of surgeons’ experience, the operation time of laparoscopic gastrectomy would significantly decrease.

Besides these findings, the prognostic significance of LNR among GC patients in our study was also investigated, results of which were elaborated as follows. Firstly, patients with high LNR were proven to have worse OS and DFS. Then, it was also demonstrated that how the surgery was performed did not significantly affect the prognosis of GC patients with low LNR or high LNR. Thus, LNR is a useful prognostic biomarker for GC patients, which is consistent with previous findings. Zhu J et al. reported that LNR was a useful predictor for survival of GC patients [26]. Wohnrath DR also studied the prognostic significance of LNR among GC patients, revealing that LNR was an independent prognostic factor for GC patients [27]. Similar results were also demonstrated in a study by Nakamura et al. [28]. Thus, combining the results of our study and previous studies, we could conclude that LNR is a useful prognostic marker for GC patients.

The limitations of the present study are worth being discussed. Firstly, the present study was a retrospective one, compromising the suggestive ability of our study. However, the conclusion of this study was consistent with a few multicenter randomized clinical trials, suggesting the credibility of this research. Secondly, the number of patients included in this study was relatively small as our center was a small-volume one. Thirdly, we did not fully compare laparoscopic surgery with open operation in terms of short-term outcomes. However, we could still say that laparoscopic GC surgery was significantly superior to open surgery in terms of short-term outcomes as this superiority has been repeatedly confirmed by multicenter randomized clinical trials among both early stages GC and locally advanced GC. Fourthly, the time span of the present study was quite long due to the relatively small volume of our center.

## Conclusions

For GC patients, laparoscopic gastrectomy plus D2 lymphadenectomy is related with at least similar long-term outcomes to open surgery and significantly better short-term outcomes than open surgery. LNR is a useful prognostic marker for GC patients.

## Data Availability

The data analyzed in this study were available from the corresponding authors on reasonable requests.

## Abbreviations

GC: Gastric cancer OS Overall survival DFS Disease-free survival MDT Multidisciplinary teamwork KLASS Korean Laparoendoscopic Gastrointestinal Surgery Study PET-CT Positron emission tomography–computed tomography OG Open group LG Laparoscopic group HR Hazard ratio CI Confidential interval CEA Carcinoembryonic antigen NORLN Number of retrieved lymph nodes NOMLN Number of metastatic lymph nodes PHS Postoperative hospital stay LNR Positive lymph node ratio
